# Supplementary material for: Comparative multiomics analysis of cell physiological state after culture in a basket bioreactor
Source: Sci Rep. 2022 Nov 23;12:20161. doi: 10.1038/s41598-022-24687-4 (PMC9686226; doi:10.1038/s41598-022-24687-4)
Supplement: Supplementary file 1 — Supplementary Information 1. [file 41598_2022_24687_MOESM1_ESM.zip › raw data/Proteomic raw data/6.KEGGenrichment/EG.vs.CG/ALL/pathway/src/00760.html]

map00760


- K01081, K19970, K03787, K08723, K08693, K11751, K20881, 3.1.3.5, R02323"

- Down regulated proteins


A0A0D9RXR7(Description:Ecto-5-nucleotidase;Gene:NT5E;log2FC:-1.6427)

Close
